# A Scoping Review on Carotenoid Profiling in *Passiflora* spp.: A Vast Avenue for Expanding the Knowledge on the Species

**DOI:** 10.3390/molecules29071585

**Published:** 2024-04-02

**Authors:** Marina de Macedo Rodrigues Leite, Daniele Bobrowski Rodrigues, Raquel Brison, Fernanda Nepomuceno, Maria Lua Bento, Lívia de Lacerda de Oliveira

**Affiliations:** 1 Department of Nutrition, University of Brasília (UnB), Campus Darcy Ribeiro, Brasilia 70910-900, DF, Brazil; marinaleite.doutorado@gmail.com (M.d.M.R.L.); raquelbsbrison@gmail.com (R.B.); fernanda.vinhal@hotmail.com (F.N.); liviadelacerda@unb.br (L.d.L.d.O.); 2 Centro de Investigação de Montanha (CIMO), Instituto Politécnico de Bragança, Campus de Santa Apolónia, 5300-253 Bragança, Portugal; 3 Laboratório Associado para a Sustentabilidade e Tecnologia em Regiões de Montanha (SusTEC), Instituto Politécnico de Bragança, Campus de Santa Apolónia, 5300-253 Bragança, Portugal; 4 Department of Pharmacy, University of Brasília (UnB), Campus de Ceilândia, Brasilia 72220-275, DF, Brazil; mldrb42@gmail.com

**Keywords:** *Passiflora*, carotenoid profile, carotenes, xanthophylls, HPLC-DAD-MS

## Abstract

The *Passiflora* genus is recognised for its ethnopharmacological, sensorial, and nutritional significance. Yet, the screening of its dietary and bioactive molecules has mainly targeted hydrophilic metabolites. Following the PRISMA-P protocol, this review assessed the current knowledge on carotenoid composition and analysis within *Passiflora*, examining 968 records from seven databases and including 17 studies focusing on carotenoid separation and identification in plant parts. Those publications originated in America and Asia. *P. edulis* was the most frequently examined species of a total of ten, while pulp was the most studied plant part (16 studies). Carotenoid analysis involved primarily high-performance liquid chromatography separation on C_18_ columns and detection using diode array detectors (64.71%). Most studies identified the provitamin A β-carotene and xanthophylls lutein and zeaxanthin, with their geometric configuration often neglected. Only one study described carotenoid esters. Besides the methodology’s insufficient description, the lack of use of more accurate techniques and practices led to a high risk of bias in the carotenoid assignment in 17.65% of the articles. This review highlights the opportunity to broaden carotenoid studies to other species and parts within the diverse *Passiflora* genus, especially to wild, locally available fruits, which may have a strategic role in enhancing food diversity and security amidst climatic changes. Additionally, it urges the use of more accurate and efficient analytical methods based on green chemistry to better identify *Passiflora* carotenoids.

## 1. Introduction

The genus *Passiflora* is widely distributed in tropical and subtropical regions, with more than 500 species already identified in Central and South America, of which roughly 150 are native to Brazil and approximately 60 produce edible, morphologically diverse, and sensorially singular fruits called passion fruits [1,2,3,4]. Passion fruits from autochthonous and exotic varieties are regionally consumed in fresh form or in very traditional preparations, such as juices, and varied culinary recipes, besides processed products [5].

Benefits for human health have long been reported after consumption of different botanical parts of the *Passiflora* plants or their products. Sleep modulation and anxiolytic effects described in folk medicine and associated with administering leaf and flower infusions from *Passiflora* spp., primarily *P. incarnata* and *P. alata*, have been more recently attributed to their natural products such as β-carboline (harmala) alkaloids and flavonoids such as chrysin and vitexin, as well as benzoflavones [6,7,8]. In the context of herbal medicine, the use of aerial parts of *P. incarnata* L. for anxiety relief and control of mild insomnia has been documented in the Brazilian Pharmacopoeia [9], with the Medicines & Healthcare Product Regulatory Agency from the UK [10], among others, also contemplating *Passiflora* applications to human health [6].

Despite the well-recognised ethnopharmacological relevance of *Passiflora* spp., the nutritional, functional, and technological potentials of some species are still considered underexploited, especially when it comes to the wild ones [4]. Among the studies with these species, Duarte et al. [11,12] reported post-prandial (3 h) and chronic (for 14 days) health benefits associated with the respective consumption of *P. setacea* pulp juice (150 g pulp) and pulp (50 g) by overweight individuals. A reduction in HOMA-IR and insulinemia, an increase in HDL, as well as modulation of genes related to inflammatory mechanisms were noticed in the former study [11], while reduced IL-6 levels were observed in the latter [12]. Additionally, Santos et al. (2021) [1] evaluated the composition of bioactive dietary components in *P. tenuifila* and reported that this fruit is a good source of fibre, phenolic compounds, and carotenoids, suggesting interesting nutritional and functional properties. Indeed, the positive effects observed in epidemiological and intervention studies from the consumption of fruits such as passion fruit have been attributed to the presence of nutrients such as vitamins, minerals, and fibre in these foods and to their phytochemical composition [13,14].

The composition of phenolic compounds in *Passiflora* genus has been reviewed [4], highlighting the characteristic presence of *C*-glycosylflavones such as vitexin, isovitexin, orientin, and isoorientin [4]. Moreover, the peel and pulp of several varieties of passion fruits present colouration suggestive of the presence of carotenoids, but the lipophilic portion of components of *Passiflora* organs, especially carotenoids, overall do not receive as much attention as the hydrophilic fraction, as can be noticed in recent reviews convening data on the chemical characterisation of this plant [15,16,17,18].

Carotenoids constitute a family of more than 700 lipophilic compounds imparting yellow, orange, and red hues to a wide range of plant tissues, in which they play primary roles as part of the photosynthetic apparatus, especially in leaves, as well as secondary functions as antifungal agents and flavour components [19,20]. Structurally, they are characterised by a hydrocarbon backbone containing a series of conjugated double bonds (cdb, commonly in the more stable (all-*E*)-configuration) and can be classified as carotenes and xanthophylls, the latter presenting oxygenated groups in their molecule in contrast to the former. Hydroxylated xanthophylls can be naturally found esterified with fatty acids to constitute the carotenoid esters, which are often neglected given the increased complexity in analysis [21].

The carotenoid class is well known due to the provitamin A activity of some of its members, primarily the (all-*E*)-β-carotene, which displays the structural requirements to be bioconverted into two molecules of this indispensable nutrient after consumption. Furthermore, beneficial health effects of the dietary intake of provitamin A and non-provitamin A carotenoids have been reported and comprise particularly the decreased risk of developing age-related macular degeneration and other eye conditions [22,23], some types of cancer [24,25], and cardiovascular [26,27] and bone diseases [28]. Humans depend on their diet to obtain carotenoids and consequently be exposed to these health-promoting activities, with the chemical structure of these compounds determining their physicochemical properties; their behaviour through digestion, absorption, metabolism, and distribution; and ultimately, their biological efficacy [29,30].

Building on the above, it is relevant to evaluate individual carotenoids in different parts of *Passiflora* plants. The information on the chemical species present may be catalogued in databases and guide nutritional strategies, as well as be the basis for epidemiological research that seeks to elucidate the association between the consumption of phytochemicals and health outcomes. The carotenoid composition analysis is also the first step in bioavailability studies focusing on better retaining the health properties of these compounds. The knowledge about the main carotenoids present in *Passiflora* plants can further promote agronomic strategies for a greater production or accumulation of these compounds by the plant [31], as well as stimulate the consumption of locally available species as a source of bioactive compounds. Therefore, this study aimed to compile the available scientific data about the composition of carotenoids in organs of *Passiflora* plants, also providing a picture of the current scenario of the main methods of extraction, separation, identification, and sometimes quantification applied to this end.

## 2. Results and Discussion

### 2.1. Search Results

**Figure 1:**
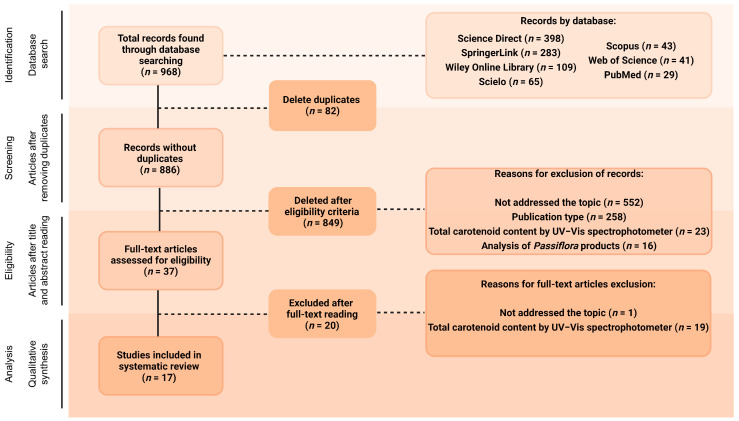
Flow diagram of the search strategy. Created with BioRender.com.

The database search, using different combinations of the chosen keywords, was blindly conducted by three of the co-authors and found a total of 968 documents (Figure 1). All the documents were arranged in the software Rayyan^®^ [32] to perform the posterior selection stages. After removing duplicates (*n* = 82), titles and abstracts of the remaining 886 articles were blindly read by three of the co-authors, and 849 of them (95.82%) were excluded for not meeting the inclusion criteria. Of these, 552 articles (65.02%) were excluded for not addressing carotenoid composition analyses, and 258 (30.39%) for consisting of publication types not considered in this review, such as review articles, conference abstracts, case studies, editorials, letters or brief communications, and book chapters. Another 23 studies (2.71%) were excluded at this stage for offering only analysis of the total carotenoid content of the crude extracts without previous separation, while 16 papers (1.88%) were not included since they analysed several products obtained from parts of *Passiflora* plants, not the parts themselves. Therefore, 37 articles were selected for full-text reading, and 17 of them were included in this review (representing 4.18% and 1.92% of the initial number of articles, respectively, not considering the duplicate ones, Figure 1). Of the 20 articles excluded at the full-text reading stage, 19 analysed only total carotenoids, and one study focused on the functional groups found in carotenoids rather than the individual identification.

First, the limited number of studies reporting the carotenoid profile of plant tissues of the numerous and widely distributed *Passiflora* genus draws attention. This subject has been considerably less explored than the phenolic compounds in the same botanical genus, as reviewed elsewhere [4]. For example, Gadioli et al. (2018) [4] found initially 2066 studies in their systematic review on phenolic compounds in different parts of *Passiflora* species, more than twice the number of studies found in the initial research of the present study (*n* = 886), and 82 out of those had, in fact, identified the polyphenols, against 17 studies presenting the carotenoid assignment included in the present review. As mentioned before, previous reviews on chemical composition of *Passiflora* plants indeed contain several papers that focus on hydrophilic fractions of compounds and relatively few ones with carotenoid analysis, most of which present only total carotenoid content or their analysis of products prepared from the plant parts rather than the plant organs themselves [15,16,17,18]. The same can be noticed in reviews summarizing the chemical composition of other plant species that included a section on carotenoids, which also reported few studies presenting the carotenoid profile of their samples, such as for buriti (*Mauritia flexuosa*) [33] and mango (*Mangifera indica* L.) [34], fruits with an expressive content of carotenoids. 

In fact, many times researchers report the total content of carotenoids in their complex plant extracts as measured spectrophotometrically, using absorption coefficients of a single carotenoid present in the mixture. Reasons for choosing this method include either its relative simplicity and rapidity compared to chromatographic or other more complex analyses or limited expertise in these particular analytical procedures, constraints in equipment and supplies, or because such an estimate is considered satisfactory for accomplishing the study’s objectives. Markedly, 42 articles have been excluded in different stages of this review process by presenting only the total carotenoid content. In other words, only one-third of the articles that aim to analyse carotenoids in *Passiflora* present the individual carotenoid profile and in some cases, quantitation. While the estimation of total carotenoid content may remain a good response in specific scenarios, such as breeding experiments and for screening purposes, it is no longer sufficient for a broader scope of carotenoid research [35]. The characterisation of the bioactive compounds in plants not only expands the knowledge into its composition but also provides key information for a more comprehensive elucidation of the mechanisms of action underlying the post-prandial and chronic biological activities observed with its intake [4].

#### 2.1.1. Time Period Covered in Papers Included and Their Origin

**Figure 2:**
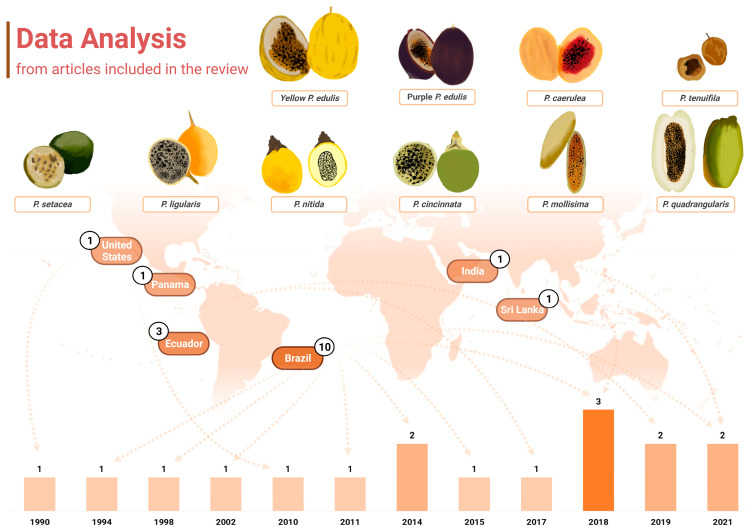
Graphical representation of the data extracted from the articles containing carotenoid identification in *Passiflora*. The distribution of publications over the years is displayed at the bottom of the figure, where the years with higher numbers of publications are indicated by progressively darker bars. Additionally, the countries of origin of these studies are displayed, along with their respective publication counts. The fruits from the various *Passiflora* species analysed in the included studies are depicted through freehand digital illustrations by co-author M. L. B., created using Picsart^®^ software (version 24.3.3). Created with BioRender.com.

Another point of interest when considering the chemical profiling of *Passiflora* samples is that almost half of the papers included in this review, which included papers between 1990 and 2021, were published in the last 5 years (*n* = 7, 41.17%), notably in 2018 (*n* = 3, 17.65%) (Figure 2). Roughly 70% of the studies were published within the past decade, which may also reflect the technological development of analytical tools and research, as well as more wide distribution of chromatographic equipment and material, even in developing countries to which several *Passiflora* species are native. Further information about the techniques applied in the studies included will be discussed later in a dedicated section. Over the years, there has been also a growing interest in studying non-commercially explored species within the *Passiflora* genus, as well as other parts of *Passiflora*, including fruit peels and leaves, which is also addressed further in this review.

It is also noteworthy that the publications included in this review were conducted in only six distinct countries across America and Asia (Figure 2). Interestingly, up to 2018, research on carotenoid identification in *Passiflora* organs was exclusively published by groups in America. Brazil was the leading contributor, accounting for 58.82% (*n* = 10) of the studies, followed by Ecuador, which represented 17.65% (*n* = 3) of the total. The United States, Panama, Sri Lanka, and India contributed one study each (5.88% of the total publications for either country). All the samples analysed were cultivated in the respective countries of publication. 

Brazil is the largest producer and consumer of passion fruit worldwide [36], with a production of 697,859 tons of this fruit in 2022 [37]. In addition, at least 150 species of passion fruit are native to this country, factors that may be related to the greater interest in characterising their fruits. Two publications from Brazil are linked to a national research network called Passitec, and they used sample fruits from the experimental field of the Brazilian Agricultural Research Corporation (Embrapa-Cerrados, Planaltina, DF, Brazil), associated with this network. In recent years, Passitec has developed research with native and wild species of *Passiflora* spp. to establish their production chain and enable the insertion of these fruits from Brazilian biodiversity in the market. This network has the largest genetic collection of *Passiflora* spp. in the world, including varieties that have been associated with functional and medicinal effects [38]. The results obtained from research carried out within the framework of Passitec have already resulted in adaptable varieties being available in the market, including *P. setacea* BRS-PC (“Pérola do Cerrado”), *P. cincinnata* va BRS-SF (“Sertão Forte”), and *P. alata* va BRS-MC (“Mel do Cerrado”) [39]. This network has been recognised as one of the most relevant bioeconomy platforms established in Brazil [40].

The second largest country in the included publications, Ecuador is the main exporter of concentrated sour passion fruit juice (from *P. edulis*, 50° brix), *P. edulis* f. flavicarpa (yellow passion fruit) being the most cultivated species in this country, as it is in Brazil and Peru [41], while in South Africa and Australia, the purple passion fruit (*P. edulis* f. edulis) stands out in production [42,43]. In Colombia, the neighbouring country of Ecuador, besides *P. edulis*, five other species are grown commercially, namely *P. ligularis*, *P. tripartita*, *P. edulis* f. edulis, *P. maliformis*, and *P. quadrangularis* [42,44], but no study has been included from this country.

#### 2.1.2. Passiflora Species and Parts Studied

This review encompassed analyses of then ten different species or varieties belonging to the *Passiflora* genus, namely yellow and purple *P. edulis* (sour passion fruit), *P. cincinnata*, *P. nitida*, *P. setacea*, *P. mollissima* (taxo or banana passion fruit), *P. caerulea*, *P. quadrangularis* (granadilla), *P. ligularis*, and *P. tenuifila* (passion fruits of these species are depicted in Figure 2). *P. edulis* was the most frequently studied species, as also reported elsewhere in terms of polyphenol composition [4] being analysed in 12 investigations of this review (70.59%). The yellow and purple cultivars were specified in eight and four of these studies, respectively. Studies with *P. edulis* have been published in research conducted in different countries, the USA, Brazil, Sri Lanka, and India, among the studies included in this review. Despite the great diversity of species of the *Passiflora* genus, the fruits of *P. edulis* in general are the most popular and widely distributed and consumed ones, mainly as an ingredient in juices, smoothies, jelly, jams, and sweets [45,46,47]. However, since 2011 an increase in research interest in other species in the genus has been identified, including wild and native species (Figure 2). In this context, Brazil and Ecuador stand out with publications that analyse native or exotic but still under-exploited species, such as the passion fruit from the Brazilian Cerrado (*P. setacea*), *P. tenuifila* [1], *P. caerulea* [48,49], *P. mollissima* [50,51], and *P. quadrangularis* [52].

**Figure 3:**
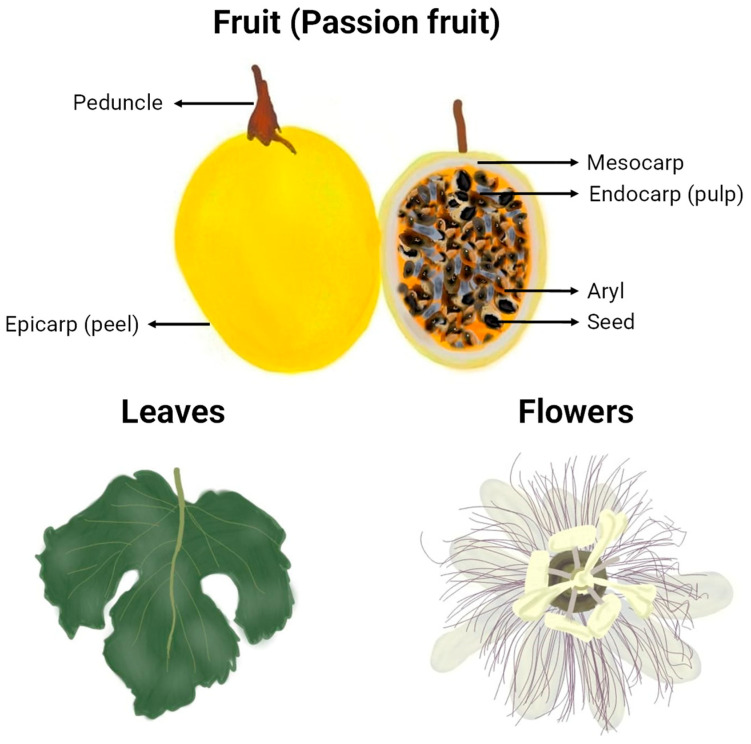
Graphical representation of botanical parts of yellow *P. edulis* (va flavicarpa). Freehand digital drawing by the co-author M. L. B. using the software Picsart^®^ (version 24.3.3).

The morphology of the passion fruits (as can be seen in Figure 2) and other plant organs varies depending on the formae, cultivar, variety, or species to which they belong. The taxonomy of the highly diverse genus *Passiflora* continues to be a subject of uncertainty, which explains why many research papers fail to identify the plant used at the infraspecific level or show inconsistency in the terminology for botanical forms of the species [16]. However, some traits are common, and an illustrative representation of general characteristics and morphological organization of yellow *P. edulis* Sims, the most studied cultivar in the papers of this review and one of the most commercialised passion fruits worldwide, can be found in Figure 3. A *P. edulis* fruit consists of a spherical to ovoid berry with a relatively tough and coloured peel (epicarp or exocarp), a middle white and spongy layer (mesocarp), and an inner peel layer (endocarp), which together are referred to as pericarp. The interior (locular cavity) is filled with the pulp, composed of numerous membranous sacs (aryl) that surround the seeds [16]. Overall, the leaves contain three to seven lobes, and the flowers are radially symmetrical with five petals and five sepals, often surrounded by a corona of brightly coloured filaments [53].

Fruit pulp was the most studied part of the plant in terms of carotenoid composition. Only one study investigated carotenoids in leaves of *P. edulis* [54]. Peels were also examined in one study that analysed samples of *P. caerulea* and yellow and purple *P. edulis* [49]. In the case of peels of *P. edulis* fruits, colours in the spectrum from pale yellow to deep purple can be observed, so documenting this trait is absolutely relevant when it comes to the characterisation of natural pigments. The whole fruit of *P. tenuifila*, including peels, seeds, and pulp, was the material used in one study as reflects the way this fruit is commonly eaten. Three studies [51,52,55] denominated their sample material as “edible parts” of passion fruits (*P. edulis*, *P. ligularis*, *P. quadrangularis*, and *P. mollissima*), and although they did not specify it, it is reasonable to assume that seeds were included as they are commonly consumed along with the pulp. 

### 2.2. Methods and Techniques Employed for Carotenoid Analysis in Passiflora

When investigating the carotenoid composition, it is critical to tailor the analytical procedure to the specific sample under investigation, adapting the protocol to its carotenoid composition as appropriated [35]. The selection of a proper method must also align closely with the objectives of the analysis. The analytical methods and approaches already adopted to examine the carotenoid composition in parts of *Passiflora* plants, along with their respective conditions, are summarised in Table 1. The sampling/preparation of the analytical sample is also of utmost importance and was considered in the quality analysis is explained in further sections.

#### 2.2.1. Carotenoid Extraction and Pre-Chromatographic Methods

Except when a direct method that does not require sample preparation is applied, carotenoid extraction is essential for their identification and quantification in foods and may affect the composition of those compounds as described in studies. This is because the final composition reported depends on whether the extraction was exhaustively performed with one or a sequence of solvents of different polarities or not, on the characteristics of the extraction solvent and how it can impact the stability of the compounds or promote the formation of extraction artifacts, on the extraction method efficiency and validation parameters, on the application of certain procedures used during the extraction such as the saponification, and on the adoption of precautions to avoid errors.

Since carotenoids are lipophilic molecules, organic solvents like acetone, hexane, ethyl acetate, and methanol are generally well suited for their extraction. Among the studies included in this review, 12 (70.59%) used acetone as the solvent in the extraction of carotenoids from: edible parts (1 fresh and 1 freeze-dried; *n* = 2); pulp (fresh; *n* = 7); pulp and peel (fresh; *n* = 1); leaves (freeze-dried; *n* = 1); and whole fruit (freeze-dried; *n* = 1). Acetone is generally the preferred organic solvent for extracting carotenoids, especially from fresh vegetable tissues, as its miscibility with water, besides the affinity for lipids, facilitates enhanced penetration into the sample, improving extraction efficiency, with satisfactory recoveries of both carotenes and xanthophylls [65,66]. However, carotenoids may be unstable in acetone, and a partitioning with a washing step is recommended to remove this solvent [66]. All those 12 studies carried out the partitioning step, transferring the carotenoids extracted with acetone to other organic solvents, with water washing to remove acetone residues. Only 3 [1,52,54] out of those 12 studies extracted carotenoids from freeze-dried samples using acetone, none of them describing whether the sample was reconstituted to its original moisture before the extraction or not. Carotenoids from freeze-dried samples are often extracted with solvents other than acetone as there is no need for a water-miscible solvent and also to avoid the partitioning and washing steps, as they are labour-intense, time consuming, and more prone to losses and errors as the analytical steps increase [66,67].

Another study was conducted using extraction from reconstituted freeze-dried pulp, applying a sequential extraction using a 1:2 mixture of hexane:NaCl (saturated sodium chloride solution in water), dichloromethane, and ethyl acetate, separately [61]. NaCl solutions can be used to prevent the formation of emulsions in the interface of organic and aqueous phases, with the higher ionic strength in the aqueous fraction favouring the migration of the extracted carotenoids to the organic fraction [68]. Also, the use of more than one solvent in sequence during carotenoid extraction is a strategy often employed to cover a wider range of polarity, which consequently allows carotenoids of different hydrophobicity levels to be solubilised and extracted, from the highly hydrophobic carotenoid esters and carotenes to the less hydrophobic xanthophylls during extraction [67,69]. However, analogous to what has been reported for samples of citrus pulp, in carotenoid analysis of passion fruit pulps, the combination of extracts from solvents of varying polarities, such as EtOAc and MeOH, may result in the precipitation of water-soluble substances, such as polysaccharides including pectin from the juice vesicle membranes, the aryls [70]. This would require a liquid–liquid partition or other separation step to remove the precipitates without losses of carotenoids, but such interference has not been observed or reported in any of the studies included in this review.

Two studies used methanol, pure [63] or in a solution with water (60:40, *v*/*v*), plus dichloromethane [50] as an extraction solvent for carotenoids from fresh passion fruit pulp. Methanol, as well as ethanol as a greener and safer option, are other examples of water-miscible organic solvents used to extract carotenoids with good penetration into the plant matrix, although there are concerns regarding the recovery of more hydrophobic molecules such as carotenes [35,71]. Still, from *Passiflora* pulp in natura, one study used chloroform [51], and one reported the use of hexane [56], low-polarity solvents that, in their turn, may not dissolve xanthophylls efficiently [35].

An important consideration is the toxicity of volatile organic solvents commonly used in carotenoid extraction, both to the analysts and to the environment. Consequently, there has been a shift towards the development and use of alternative, greener extraction methods and solvents [72,73]. These innovative approaches have also demonstrated good performance in carotenoid recovery and final extract quality in terms of bioactivities, showcasing their potential to improve analytical outcomes [74,75]. For instance, supercritical fluid extraction has been successfully applied in the extraction of carotenoids from orange peel [75]. Another strategy to eliminate the use of organic solvents involves the production of carotenoid-rich oils, as in the study by Chutia and Mahanta (2021) [70], which applied olive and sunflower oils as solvents in the extraction of carotenoids, assisted by ultrasounds and microwaves, from purple passion fruit peel. However, these studies, focusing, respectively, on carotenoids of a *Passiflora* residue and on the total carotenoid content, were not included in this review as no included study employed such innovative extraction techniques. Clearly, the use of innovative solvents and techniques, compatible with green chemistry, is a trend that demands more attention and application in *Passiflora* research. 

A critical aspect in carotenoid extraction is avoiding an acidic environment or acid contact, so the natural acidity of passion fruit pulp may pose a challenge in carotenoid analysis of its matrix. Ramaiya et al. (2013) [76] measured the pH of pulp from various *Passiflora* species grown in Malaysia, and the values ranged from 3.16 to 3.76, while the total titratable acidity ranged from 0.88% to 3.03%. Citric acid was found to be the main organic acid present in *P. edulis*, *P. maliformis, P. quadrangularis*, and *P. tenuifila* fruits [1]. In Brazil, the standard of identity and quality for passion fruit pulp is pH = 2.7, and total acidity in citric acid is 2.5 g/100 g in fresh weight (fw) [77]. The release of acids from plant tissues and the resulting increase of the acidity of the medium during the extraction may promote *E*/*Z* isomerization of the carotenoids present in the sample, impacting the native carotenoid profile; thus, the use of a neutralizing agent can be useful to prevent this from occurring [67]. Interestingly, among the studies we reviewed, only one [55] utilised NaHCO_3_ to neutralise the organic acids in the sample.

Moreover, during the carotenoid extraction procedure, a clean-up step of saponification is traditionally employed to remove interfering compounds, such as triacylglycerols and chlorophylls, simultaneously extracted from plant matrices. This procedure was carried out in 11 (64.71%, [2,48,49,51,52,55,56,58,59,60,62]) of the 17 studies included. A 10% (*w*/*v*) solution of KOH in methanol (*n* = 7, 63.64%, [2,48,49,52,58,59,60]) and a saturated ethanolic solution of KOH (*n* = 2, 18.18%, [56,62]) were applied, in both cases, with an overnight duration. The other two studies used a 5% (*w*/*v*) methanolic solution of KOH, with durations of 4 h at 50 °C [51] and 2 h at room temperature [55]. Whereas this procedure is more commonly carried out with the non-saponified carotenoid extract [2,48,49,51,52,55,58,59,60,62], some research groups perform it with the homogenised plant matrix before the addition of extraction solvents [56], the vegetal tissues being likely softened in this procedure, which might facilitate the extraction. Although producing a cleaner extract, saponification increases the extraction time and may lead to the degradation of carotenoids, especially lutein and violaxanthin, as well as the formation of extraction artifacts [66]. Hot and long saponification procedures and those carried out with high concentrations of alkali may intensify such reactions, which can result in a misleading final interpretation of the qualitative and quantitative data [78]. As the impact of the saponification procedure on the quality of the saponified extract obtained and extraction efficiency depends also on the sample matrix and the type of carotenoid species present, preliminary tests and method validation are recommended to balance variables such as time, temperature, and alkali concentration and define the mildest conditions sufficient to hydrolyse the esters, which can be monitored in a straightforward way by TLC, and then by HPLC [79,80]. In addition, when the sample presents carotenoid esters in its composition, the ester bonds between xanthophylls and fatty acids are also cleaved during saponification. Although this is a choice made by several researchers because the complexity of analysis is reduced, the information on the native carotenoid profile of the sample is also lost, and the relatively new notion that the esterification may impact the bioavailability of carotenoids make this examination of relevance in carotenoid research. The six remaining studies (35.29%) [1,50,54,57,63] did not perform the saponification step, but only one [1] identified and quantified carotenoid esters in its *Passiflora* sample.

#### 2.2.2. Carotenoid Separation and Identification

All separation and identification strategies applied by the studies reviewed are also summarised in Table 1. Carotenoid separation is traditionally performed by using liquid chromatography techniques, which was true for the set of papers included in this review. Among them, carotenoid separation was achieved by using thin-layer chromatography (TLC, *n* = 1), open column chromatography (OCC, *n* = 2), high-performance liquid chromatography (HPLC, *n* = 15), and its fast and more efficient variants ultra-fast liquid chromatography (UFLC, *n* = 1; 5.88%) and rapid-resolution liquid chromatography (RRLC, *n* = 1; 5.88%), employed alone or combined. Two studies [57,58], both from the 1990s, performed an OCC procedure, associated with TLC or not, before injection into HPLC.

Godoy and Rodriguez-Amaya (1994) [57] investigated the presence of (*Z*)-isomers of provitamin A carotenoids in several Brazilian fruits, including *P. edulis*. To do so, they performed a two-step OCC separation, first on a MgO (magnesium oxide)/Hyflo Supercel (Celite^®^, a form of diatomaceous earth, SiO_2_) phase, where they achieved the separation of the provitamin A carotenoids, followed by a calcium hydroxide (Ca(OH)_2_) column to separate the carotenoid isomers. To identify the carotenoid profile of yellow *P. edulis*, Mercadante, Britton, and Rodriguez-Amaya (1998) [58] applied the separation using OCC containing neutral alumina (a treated aluminium oxide—grade III) having separated the carotenoids into three fractions according to their polarity (i. carotenes and epoxy-carotenes; ii. monohydroxy- and keto-carotenoids; iii. polyhydroxy-carotenoids). Then, each band was re-chromatographed by TLC on silica gel, and the bands obtained were again purified by TLC (MgO/Kieselguhr—a form of diatomaceous earth). TLC is a chromatographic separation technique well suited for qualitative analysis and is the method performed in the latter study since the final recovery of carotenoids may not be quantitative [69].

Among the normal-phase stationary phases employed in those classical liquid chromatography studies, silica gel separates carotenoids mainly according to their polarity (the less non-polar carotenoids show higher retention), while MgO presents different adsorption affinities depending on the number and arrangement of cdb in the molecule, with carotenoids with a higher number of cdb showing greater retention [68]. Hyflo supercel^®^ also helps to control adsorption of compounds in MgO, preventing polar carotenoids from irreversibly adhering to the column [66]. On the other hand, chromatography on Ca(OH)_2_ is widely used and necessary for separation of the isomers, which would not be achieved using the previous columns. Those methods applied by both studies were considered the best way to separate and study carotenoid isomers in combination with HPLC using a reversed phase C_18_ column at that time, when a C_30_ column was not available yet.

Regarding the analysis in liquid chromatographs, studies by García-Ruiz et al. (2017) [50] and Gunathilake, Ranaweera, and Rupasinghe (2018) [54] used RRLC and UFLC equipment, respectively. García-Ruiz et al. (2017) [50] performed the chromatographic run of their carotenoids in 12 min, which indeed represents important time savings compared to the studies that used HPLC equipment analysed in this review. The average run time in studies employing HPLC was 57.22 min (nine studies reported this information), reaching up to 130 min. Gunathilake, Ranaweera, and Rupasinghe (2018) [54] did not report the total run time of their samples. As for the resolution quality of the chromatograms, we cannot compare it with other studies present in this review as they were not available in the respective publications. However, Stinco et al. (2014) [81] developed and validated the method applied by García-Ruiz et al. (2017) [50], demonstrating that there was good quality of separation of nine carotenoids present in their samples.

The LC stationary phases more commonly applied to separate carotenoids are reverse-phase C_18_ and C_30_ columns [82]. In this review, 11 studies (64.71%, [2,50,51,52,55,56,57,58,59,62,63]) used a C_18_ column for carotenoid separation, one [58] of them also using a nitrile column for xanthophyll separation; five studies (29.41%, [1,48,49,60,61] applied a C_30_ column; and one study used a C_8_ column (5.88%, [54]. The development and use of the C_30_ column to separate carotenoids is relatively recent, with the first study being published in 1994 [83], so studies before this data, such as those of Homnava, Rogers, and Eitenmiller (1990) [56] and Godoy and Rodriguez-Amaya (1994) [57], did not have this option at that time. This column was developed to specifically meet the demands corresponding to carotenoid analysis and allowed for more efficient separation of geometric and structural isomers in contrast to the C_18_ column, as well as superior selectivity for both polar and non-polar carotenoids [84,85]. Whereas the retention time of carotenoids in HPLC analyses is influenced by multiple factors related to mobile and stationary phases, such as the composition of solvents and temperature in which the column is kept, overall studies have been demonstrating higher relative retention times of these compounds in C_30_ columns in contrast to C_18_ phases, related to the higher hydrophobicity of the former (longer C-chain) [86,87,88]. Moreover, due to the differences in retention capacities, it is also possible to observe certain changes in the elution order of some carotenoids when the separations are carried out on C_18_ or C_30_ columns, since retention occurs basically according to polarity in the C_18_ column, and in the C_30_ column other factors such as the size of the molecule, the polyene chain length, and hydrophobicity are also to be considered [89].

Additionally, the solvents used in the mobile phases and the C_30_ columns are usually more expensive and selective, which may lead to the use of the C_18_ column in several studies [82]. Column temperature is another relevant factor to be considered in combination with other method parameters in the separation of carotenoids by HPLC, since it influences the selectivity and the retention time of carotenoids and may lead to biases in the interpretation of results [66]. Among the 17 studies included in this review, only 6 specified the temperature applied on the column during the separation of carotenoids, which ranged from 22 °C to 33 °C. The management of column temperature during carotenoid separation was discussed in the review by Rivera and Canela-Garayoa (2012) [85].

Several organic solvents and their combinations can be used to compose the mobile phases applied in HPLC separation of carotenoids. In the studies included in this review, eight different solvents were employed as mobile phase constituents, namely: acetonitrile (ACN) (*n* = 8; 40.06%), dichloromethane (CH_2_Cl_2_) (*n* = 3; 17.65%), methanol (MeOH) (*n* = 17; 100%), ethyl acetate (EtOAc) (*n* = 5; 29.41%), hexane (*n* = 1; 5.88%), methyl tert-butyl ether (MTBE) (*n* = 5; 29.41%), isopropanol (*n* = 2; 11.76%), and water (*n* = 4; 23.53%). MeOH was used in all studies included in this review, regardless of the stationary phase. All five studies that used a C_30_ column as stationary phase applied MeOH and MTBE as mobile phases. Three of these studies added water to their mobile phases, and one added 0.1% triethylamine (TEA) to the MeOH:MTBE solution; the latter was also applied in two other studies using C_18_ columns. TEA is an organic compound used at low concentrations (due to possible pH changes) to compose mobile phases in carotenoid analysis to improve symmetry and reduce peak tailing, as it influences compound retention [87].

The LC detectors used in the carotenoid identification of *Passiflora* parts comprised those traditionally employed in carotenoid research. These comprise detection based on UV–Vis light absorption, either at a specific wavelength (UV–Vis detector) or across a wavelength range (diode array detector, DAD), and the more sensitive mass spectral detection in mass spectrometers (MS) to obtain insights on the compounds’ molecular mass. Five studies (29.41%) [48,49,62,63] reported the use of UV–Vis detectors, thirteen (76.47%) [1,2,50,51,52,54,55,56,57,58,59,60,61] used DAD, and only three (17.65%) [1,58,60] employed MS, in series. Four studies [48,49,62,63] applied only UV–Vis detectors, and Homnava, Rogers, and Eitenmiller (1990) [56] applied DAD, in addition to UV–Vis, only for the purple *P. edulis* samples.

The identification of carotenoids that have been separated by chromatographic techniques was generally performed according to parameters of the chromatographic elution in combination with spectral characteristics of each peak provided by UV–Vis, DAD, and MS detectors (Table 1). The retention time on a reverse-phase column under the applied chromatographic conditions, where the compounds typically elute in order of polarity along with the elution order may provide insights on the type of compound, were indeed observed by many of the authors during identification, in combination with UV–Vis (wavelength of maximum absorption, fine spectral structure; *cis* peak intensity) and MS spectral characteristics (often in positive mode, so protonated molecule ([M + H]^+^); and MS/MS fragments). The combination and interpretation of this information offer important structural clues on the carotenoid molecule and allow comparison with authentic standards or with data present in the scientific literature for the identification of the carotenoids in the sample, especially those also using more accurate and orthogonal responses such as nuclear magnetic resonance spectroscopy (NMR) and molecular dichroism, which remain not frequently used in laboratory routines.

Mercadante, Britton, and Rodriguez-Amaya (1998) [58] applied NMR to identify prolycopene, a molecule that cannot be properly distinguished from lycopene by only using mass spectrometry, due to the isomerization promoted by the high ionization temperature. The application of NMR in this study allowed the differentiation and the unequivocal identification of the prolycopene molecule. Oliveira et al. (2014) [61] used near-infrared (NIR) and mid-infrared (MIR) spectroscopy techniques for predicting ripening parameters in yellow *P. edulis*, including β-carotene content. In this study, it was observed that NIR and MIR techniques were not suitable for the β-carotene, and the authors justify the low concentration of these compounds compared to other components of the food. Infrared spectroscopy techniques are known for being best suited for macroconstituents (≥0.5%), and its application for the quantification of bioactive compounds in foods, including carotenoids, was recently reviewed by Johnson et al. (2023) [90]. The use of these direct, non-destructive analysis techniques would facilitate real-time sample monitoring without extensive preparation, presenting a significant advantage over traditional methods by reducing chemical and time consumption [91,92].

It is important to note that the identification of carotenoid esters remains a challenge. Besides the presence of interfering compounds in non-saponified extracts, there are two major difficulties in the carotenoid ester analysis: (1) the variety of molecules that can be formed by the various combinations of xanthophylls and fatty acids, which also increases the number of chromatographic peaks and often reduce the peak resolution and (2) the fact that there is no difference in the chromophore among the different esters of a given xanthophyll and between them and the free xanthophyll, so the utilization of either UV–Vis or DAD detectors does not provide enough information for molecule identification. In these cases, the application of at least mass spectrometry becomes indispensable to obtain a sufficient response for the tentative identification of the molecule. This technique, particularly with high-resolution analysers like QTOF and Orbitrap, plays a crucial role in the assignment of carotenoids in general as it provides more sensitive and accurate molecular mass information, assisting the structural assignment [93,94].

Furthermore, two studies applied specific chemical reactions to obtain structural information on the carotenoid molecules of their studies. Godoy and Rodriguez-Amaya (1994) [57] applied acetylation and methylation reactions of the extracts obtained to investigate the presence of hydroxycarotenoids. In both cases, the presence of the hydroxyl group was characterised by an increase in the retention factor in TLC. Reduction with NaBH_4_ was also applied to investigate the presence of apocarotenals, the positive reaction enabling the observation of a characteristic three-peak carotenoid spectrum, referring to the hydroxycarotenoid formed in the reaction. In addition, the iodine-catalysed isomerization was carried out for *E*/*Z* isomer differentiation, also in the study by Mercadante, Britton, and Rodriguez-Amaya (1998) [58]. This reaction is based on bathochromic and hypsochromic effects in the wavelength of maximum absorption for (*Z*)- and (all-*E*)-carotenoids, respectively [66].

Finally, using the techniques presented and data analysis tools, comprehensive metabolomic research may offer further insights into the formation of chemical structures such as apocarotenoids and oxidative products [95].

#### 2.2.3. Carotenoid Quantification

Whereas the analysis of carotenoid quantity was not an inclusion factor in the present review, 16 articles of the total 17 presented the individual carotenoid quantification in their samples. Most of the studies employed DAD or UV–Vis detector responses after chromatographic separation, thirteen using external [1,2,48,49,50,51,52,54,55,56,59,60,62] and one using internal standardization [61], while one study [57] estimated the concentration of isolated carotenoids with a UV–Vis spectrophotometer using their absorption coefficients. In the latter, authors stated that the quantitation was not performed by HPLC-DAD given the lack of chromatographic resolution of the geometric isomers and the difficulty in the acquisition of (*Z*)-isomer standards [57]. In one study, it was not clearly stated how the carotenoids were quantified [63], and none of the 17 carotenoids were quantified by using mass spectrometry. For the external calibration, solutions with known concentrations of carotenoid standards of high purity were prepared within the linear working range and separately injected under the same conditions as the carotenoid extract of interest [96]. Although commonly used for quantification in carotenoid analysis by HPLC-DAD, external standardization does not account for any potential losses of analytes during sample preparation and analysis. The internal standardisation, i.e., the addition of a known quantity of internal standards to the sample at the beginning of the analytical process, might help to minimise this bias and ensure greater measurement accuracy [97]. However, the choice of an internal standard can be challenging since, to guarantee the accuracy of the measurement, the compound must exhibit similar chemical properties to the carotenoid of interest, but at the same time, it must be absent in the sample matrix and not overlap carotenoid peaks during chromatographic elution [96]. The study included in this review that carried out quantification using an internal standard used β-apo-8′-carotenal [61], a naturally occurring apocarotenoid originated from the β-carotene and one of the most applied internal standards for carotenoid quantification [98,99].

Among the papers included in which external curves were applied, carotenoids were generally quantified using their specific authentic standards [48,49,50,51,52,54,55,56,59,60,62]. However, for those studies in which a broader identification was presented, standard curves of a carotenoid with a similar chromophore were applied to quantify those compounds for which a standard was not available, or the (all-*E*)-carotenoid standard curve was applied to quantify its respective (*Z*)-isomer [1,2,61]. As it is not always possible to obtain all the necessary standards, it is a common practice the use a carotenoid standard that shares UV–Vis spectral characteristics with the carotenoid of interest for quantification [96]. The same strategy is generally applied for the quantification of carotenoid esters, given the low commercial availability of their standards [100]. In this sense, external calibration curves of free xanthophylls were applied for the carotenoid ester quantification in whole fruit of *P. tenuifila* [1]. However, this is not free from bias, and under ideal conditions, the first choice should be using the specific carotenoid for quantification [35,101]. This is particularly true when considering a gradient method, since carotenoid light absorption is affected by the solvent composition, and, for instance, free xanthophylls elute in different chromatographic regions than their ester forms [96]. Other methods that have also been applied for carotenoid ester quantification include: (i) the calculation of the result as a percentage of area (area obtained from the peak of the carotenoid ester as a function of the total area of the chromatogram); (ii) the carotenoid ester standard synthesised in the laboratory; and (iii) the application of molecular weight correction factors (when utilizing a calibration curve of a free carotenoid to measure a carotenoid ester with an identical chromophore) [96,100].

Additionally, an exhaustive extraction of carotenoids was reported only in three studies [1,57,60] included in this review. When the characterisation of a sample is the purpose of the analysis, the adoption of a quantitative approach is important to guarantee the accuracy, reliability, and relevance of the findings. For an important discussion on quality assurance for carotenoid analysis, readers are referred to the work of Rodriguez-Amaya (2010) [102].

### 2.3. Carotenoid Composition of Parts of Passiflora Plants

**Figure 4:**
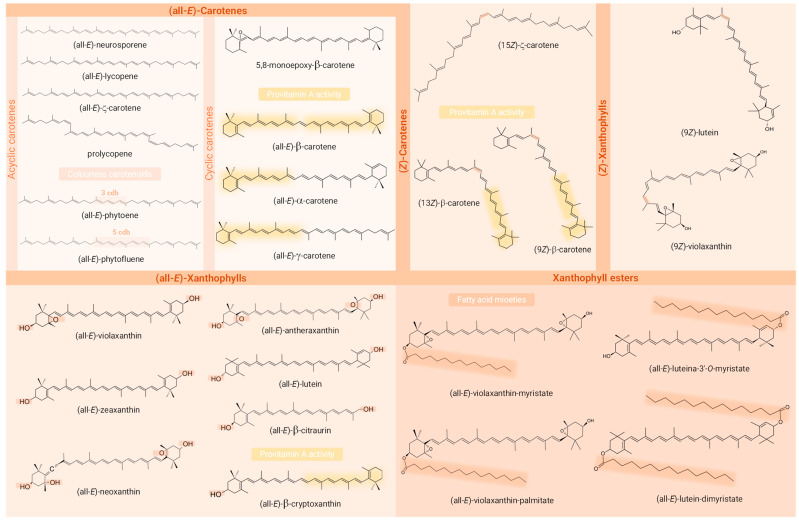
Chemical structures of the carotenoids identified in the *Passiflora* samples analysed in the studies included in the review. Structural requirements for provitamin A activity (presence of a β-ring and a C11 polyenic chain), functional groups containing oxygen, the (*Z*)-bond, and the fatty acids acylated are highlighted in the structures of provitamin A carotenoids, xanthophylls, (*Z*)-geometric isomers, and carotenoid esters, respectively. Created with ChemSketch^®^ (V14.00, FREE).

A total of 29 different carotenoid molecules were detected in different organs of *Passiflora* plants of different species, in the articles analysed (Table 2, Figure 4). This estimate is a result of the following assumptions: (i.) most of the articles included in this review did not report the geometric isomerism of carotenoids (see Table 2), and in this case, when not specified, carotenoids were computed as (all-*E*)-isomers since it is the most common form of the majority of carotenoids in nature; (ii.) (*Z*)-isomers reporting the number of the carbon with (*Z*)-unsaturation in some studies and those not reporting this number were considered once during counting, as well as cryptoxanthin and β-cryptoxanthin. Moreover, it should be noticed that 11 of the 17 studies (64.71%) included in this review were interested in identifying a fraction of the carotenoids of their samples rather than the total profile. Four studies [50,54,60,61] stated the aim of determining only two or three major carotenoids in their samples, while the study by Murillo et al. (2010) [55] examined only the macular xanthophylls lutein and zeaxanthin in passion fruits from Panama. In addition, Homnava et al. (1990) [56] and Godoy and Rodriguez-Amaya (1994) [57] exclusively identified carotenoids with provitamin A activity. Only a single study included in this review investigated and reported the whole native carotenoid profile including the xanthophyll esters [1].

Among the identified compounds, 14 were classified as carotenes, 9 as free xanthophylls, 5 as xanthophyll esters, and 1 as an apocarotenoid. An exception to the article that analysed only macular xanthophylls, β-carotene was identified in all the studies, being the only carotenoid common to all of them. Moreover, among the most frequently described carotenoids in *Passiflora* parts, the xanthophylls lutein (53% of studies), zeaxanthin, and β-cryptoxanthin (41% each) stood out, being followed by the carotenes lycopene (29%) and α- and ζ-carotene (24% each). Interestingly, whereas β-carotene, β-cryptoxanthin, lutein, and lycopene are often characteristic compounds in many fruits, ζ-carotene is more commonly detected in *Passiflora* samples, even being the major compound found in some samples. Regarding the geometrical isomers, (13*Z*)- (or (13*Z*)- or (15*Z*)-) and (9*Z*)-isomers of β-carotene were reported in three [1,2,58] and one [1] study, respectively, whereas in the article intended to identify (*Z*)-isomers of provitamin A carotenoids, they were absent in the sample analysed [57]. The compounds (*Z*)-ζ-carotene, (*Z*)-violaxanthin, and (9*Z*)- and (13*Z* or 13′*Z*)-lutein, besides an unidentified poly-(*Z*)-carotene, were also reported in these very limited studies (*n* = 4, 23.53 % of the total) included in the present review that differentiated the geometric isomers of at least one of the compounds identified, most not describing the carbon where the cis bond occurs. The identification of carotenoid constitutional and diasteroisomers can be challenging due to various factors [103]. These include the large number of possible isomers that can be found in nature and in crude plant extracts for a single carotenoid molecule, given their long system of cdb (geometric isomers) and the structural complexity of molecules within the carotenoid class. Many of these compounds have similar physicochemical properties such as polarity, which can lead to peak co-elutions. The UV–Vis spectra of mixtures often complicate the analysis of variables such as spectral fine structure (%III/II) and may preclude compound identification [104]. Another point that makes it difficult to identify carotenoid isomers is the low commercial availability of standards that cover all possible isomers of a given carotenoid. Also, the possibility of their formation as analysis artefacts can be a confusing factor in relation to the actual composition of the sample [67]. Whereas isomers sharing the same chromophore cannot be differentiated by DAD, UV–Vis spectra characteristics are generally very useful for distinguishing geometric isomers of carotenoids, the (*Z*)-forms showing a reduction in absorbance and fine spectral structure in contrast to their (all-*E*)- counterparts. In addition, the *cis* peak emerges (142 nm below the λ max), although it is not always clearly visible as the *cis* bond approaches the extremities of the molecule [105]. In the case of constitutional isomers, combining UV–Vis or DAD with MS responses provides complementary information and may allow their correct identification, since these isobaric molecules may present different absorption spectra or MS/MS fragmentation patterns despite the having same molecular weight [99,104]. Although MS provides important structural information that contributes to the identification of carotenoid peaks, it still only allows tentative identification and is unable to distinguish between *E*/*Z*-isomers and between 5,6- and 5,8-epoxides [35]. Thus, NMR stands out as a particularly useful tool for the unequivocal identification of carotenoid isomers. This technique allows analysis of the environment of each hydrogen atom using 1H-NMR spectra, as well as the type of each carbon atom using 13C spectra, enabling the identification of the precise location of the cis bonds and the full elucidation of the structures [105,106].

The only study reporting the geometric isomers of all the compounds identified with clarity was also the only study to report the carotenoid ester assignment. The analysis of the carotenoid esters and the geometry of the molecule including the configuration of cdb is of high relevance, since carotenoids with different structural forms exhibit different properties and functions including their interaction with different enzymes, and even their provitamin A activity, when this is the case [30].

Gunathilake, Ranaweera, and Rupasinghe (2018) [54] did not perform a comprehensive analysis of the carotenoids present in *P. edulis* leaves but focused only on β-carotene and lutein as they are the most common in leaves, where they play a role as primary carotenoids in the plant’s photosynthetic apparatus, and due to the important antioxidant activity of these compounds. In this study, the lutein content found in *P. edulis* leaves was the lowest among the other leaves analysed (*Sesbania grandiflora*; *Cassia auriculata*; *Gymnema lactiferum*; *Olax zeylanica*; *Centella asiatica*). Since this was the only study included that evaluated carotenoids from *Passiflora* leaves, we therefore highlight the gap in the identification of the carotenoids that constitute the leaves of *Passiflora* plants.

Out of the 12 studies analysing passion fruit pulp, 10 (83.33%) focused on *P. edulis*, including both purple [49,56,59,63] and yellow varieties [2,49,56,58,59,60,61,62], while 3 studies [54,55,57] did not specify the variety. Of these, only three provided a detailed identification of carotenoids in yellow *P. edulis* [2,58,59], with one of these studies also examining purple *P. edulis* [59]. In the purple variety, 8 carotenoids were identified [59], whereas 11 to 13 carotenoids were found in the yellow variety [58,59]. β-carotene, ranging from 2.39 µg/g to 13.35 µg/g, and the colourless phytofluene (not quantified) were the most common in both varieties across all three studies. In the other studies, β-carotene and ζ-carotene were the most abundant carotenoids of *P. edulis* pulp, in their (all-*E*)- and (*Z*)-forms [2,59]. Notably, Konta et al. (2014) [60] identified only these two carotenes as the major ones in yellow *P. edulis* pulp, with average contents of 13.8 µg/g and 16.7 µg/g (fw) for β-carotene and ζ-carotene, respectively. Additionally, two studies [2,59] reported (13*Z*)-β-carotene, varying from trace amounts to 0.40 µg/g (fw).

When comparing conventional and organic growing systems, Pertuzatti et al. (2015) [62] found β-cryptoxanthin as the major carotenoid in yellow *P. edulis* pulps, its concentration being higher in the conventional (249.90 µg/g, fw) than in the organic cultivation system (139.40 µg/g, fw). The authors attributed this result to the difference in light exposure or fruit ripening control between the two treatments analysed. In this study, β-carotene levels varied from 0.56 µg/g, fw to 0.77 µg/g, fw, which are lower than the values found in the studies reported above for the same species and organ.

Reis et al. (2018) [48,49] characterised the pulp of *P. caerulea* passion fruit and reported lycopene as the most abundant carotenoid, with values varying from 44.05 µg/g [49] to 108.39 [48] µg/g, both in dry basis. This result is consistent with the fact that this species exhibits a more reddish coloured pulp compared to other *Passiflora* species (Figure 2). In comparison to the pulp of purple and yellow *P. edulis*, passion fruit pulp from *P. caerulea* presented higher content of lutein, zeaxanthin, and lycopene, while yellow *P. edulis* showed higher content of β-carotene [49]. This study was the only one that also analysed a peel from passion fruits and found that the *P. caerulea* sample presented higher contents of all five carotenoids identified (lutein, zeaxanthin, cryptoxanthin, α-carotene, and β-carotene) [49]. In addition, lutein and β-carotene were the major carotenoids found in the passion fruit peel, regardless of the species.

Wondraceck et al. (2011) [59] analysed the carotenoid profile of pulps from different passion fruits cultivated in the Brazilian Savannah (*P. cincinnata*, *P. nitida*, *P. setacea*, native *P. edulis* (yellow), native *P. edulis* (purple) and commercial *P. edulis*). The major carotenoids varied according to the species studied, being (all-*E*)-β-carotene the predominant in *P. setacea*, (*Z*)-ζ-carotene the predominant in yellow and purple native *P. edulis* samples, whereas (all-*E*)-ζ-carotene and (all-*E*)-β-carotene were the major compounds in commercial *P. edulis* samples. These results demonstrate the variety of carotenoid composition that can be found in passion fruits, which makes the complete characterisation of the carotenoid profiles of the most diverse species of passion fruit of great relevance.

*P. mollissima* pulp [50] and edible parts [51] were evaluated in studies from Ecuador. Both studies did not perform a complete analysis of the carotenoid profile of *P. mollissima*. García-Ruiz et al. (2017) [50] reported the three major carotenoids, α-carotene (1.64 µg/g, dw), β-carotene (79.74 µg/g, dw), and zeaxanthin (1.86 µg/g, dw), while Pérez-Balladares et al. (2019) [51] reported β-carotene (16.25 µg/g, fw) and lutein (45.37 µg/g, fw). Neither of the two studies reported the moisture content of their samples; thus, considering the passion fruit moisture content around 85% [16], we can estimate the β-carotene content of *P. mollissima* pulp from García-Ruiz et al. (2017) [50] in fresh weight to be around 11.96 µg/g, which is slightly lower than that found by Pérez-Balladares et al. (2019) [51]. However, it is important to highlight that the studies might have analysed different parts, as edible parts might include seeds.

Pérez-Balladares et al. (2019) [51] also analysed edible parts of *P. ligularis* pulp, and theirs was the only study analysing this species. They found that *P. ligularis* presented lower values of β-carotene and lutein when compared to *P. mollissima* analysed in the same study. But both species presented higher values for lutein when compared to *P. edulis* (variety not specified) edible parts (0.1 µg/g, fw) [55]. Guevara et al. (2019) [52] detected only β-carotene (<0.5 mg/100 g) in edible parts of *P. quadrangularis*. Lycopene and lutein were also analysed in this study but not detected in their sample. To the best of our knowledge, no other studies have evaluated the complete carotenoid profile in *P. quadrangularis*, *P. mollissima*, or *P. ligularis*, so the composition of these species warrants further investigation.

The study of Santos et al. (2021) [1] was the only one presenting a detailed carotenoid profile. Four carotenoid esters were identified in the whole fruit of *P. tenuifila* grown in the Brazilian Savannah, which corresponded to the monoesters (all-*E*)-violaxanthin-myristate, (all-*E*)-violaxanthin-palmitate, (all-*E*)-luteina-3′-*O*-myristate, and the diester (all-*E*)-lutein-dimyristate. This study analysed ripe and mature green fruits and found that for ripe fruits the major carotenoid was (all-*E*)-β-carotene, ranging from 8.38 µg/g, fw to 11.10 µg/g, fw, values that are within the range found for other passion fruits analysed in this review. As for mature green fruits, (all-*E*)-lutein was the major carotenoid (11.86 to 13.24 µg/g, fw). The study also highlighted that the variation in the composition of carotenoids between ripe and mature green stages, especially among the two major compounds, contributed to the discrimination of *P. tenuifila* fruit according to the stage of fruit ripeness.

This review underlines the remarkable qualitative and quantitative variation in composition of carotenoids from *Passiflora* parts among the studies and among different samples analysed in the same study. This diversity is expected as it reflects the complex interaction of genetic (including species and varieties to which they belong) and environmental (cultivation conditions, climate, temperature, soil type, water availability, maturity stage, and post-harvest handling, among others) factors influencing the synthesis and accumulation of these metabolites in different vegetable tissues and organs. Plants grown in different locations, or at different times of the year, may present different carotenoid content and profile, as observed [29]. Moreover, the analytical technique employed largely impacts the magnitude and accuracy of the result. Therefore, it is of particular importance to analyse the results with caution. On the other hand, these results are a good indication that *Passiflora* organs can be further exploited as a source of carotenoids, primarily provitamin A ones.

Despite not being part of this study to evaluate different applications of *Passiflora* in industry, it is relevant to emphasise that some studies considered these applications in their analyses, which demonstrates this potential. García-Ruiz et al. (2017) [50] considered the use of *P. mollissima* as additive in the food, pharmaceutical, and cosmetic industries and applied a spray-dried technique to produce microencapsulate *P. mollissima*, which showed good stability in the bioactive compounds analysed and maintenance of their functional properties. Samyor, Deka, and Das (2021) [63] produced a foam mat-dried passion fruit powder from *P. edulis* Sims pulp and found greater content of β-carotene in the powder, demonstrating that there was good carotenoid retention even after processing. The powder produced is suitable for use in the food industry, replacing artificial additives and incorporating nutritional value into the food.

The analysis of the studies included in this review allows reinforcing the nutritional potential of edible *Passiflora* fruits, which, added to its pleasant sensory characteristics, may represent an excellent option for consumption in natura and application in the food industry. However, it is necessary to expand the studies about the comprehensive identification of carotenoids from *Passiflora*, using adequate methods that allow the good identification of the molecules present in the samples, especially when it comes to unexplored and underused wild species. Increasing knowledge about the composition of *Passiflora* carotenoids can open space for optimizing its production and make greater use of the species, resulting in increased consumption by the population (edible parts), and explore the possibilities of their application in the industry.

Carotenoids identified in *Passiflora* samples, as examined in this review, were shown to exhibit promising health benefits. Provitamin A activity was associated with carotenoids such as β-carotene, α-carotene, γ-carotene, and β-cryptoxanthin found in *Passiflora* plants, crucial for fighting vitamin A deficiency, particularly in developing countries and for people with vegetarian or vegan diets [107]. *Passiflora* extracts also contain lutein and zeaxanthin, known for promoting eye health by acting as antioxidants and protecting against blue light, showing efficacy in enhancing eye health for individuals with and without eye diseases [108]. Additionally, colourless carotenoids, phytoene and phytofluene, found in *Passiflora* plants have been associated with decreased risk of developing cancers, antioxidant activity, and skin-damage prevention [109]. Given the health potential of carotenoids described *Passiflora*, further studies that specifically address the bioactivities of these compounds from plants of this genus are encouraged.

### 2.4. Analysis of the Risk of Bias in Carotenoid Identification from Passiflora

The main purpose of this review was to verify the carotenoids already identified in *Passiflora*, although quantitative data was also included when the included study provided it. Therefore, aspects related to carotenoid identification parameters were carefully considered when analysing the risk of bias in the included studies. The proper identification of carotenoids depends on the appropriate interpretation of combined information provided by different analytical tools, so the less information available about the carotenoid structural characteristics, the less assertive the identification [110]. Minimum parameters that should be analysed for carotenoid identification are the following: “(i) UV–Vis absorption agreement with the suggested chromophore. (ii) Chromatographic data on two different systems, preferably Rf (TLC) and Rt (HPLC), including co-chromatography with an authentic sample. (iii) Mass spectrum with at least one quality allowing confirmation of the molecular mass” [65,111]. In cases where this minimum information cannot be obtained, it is recommended to use the terms characterisation or tentative identification; also, the more parameters one can combine for carotenoid determination, the more likely it is that the identification will be assertive [105].

**Figure 5:**
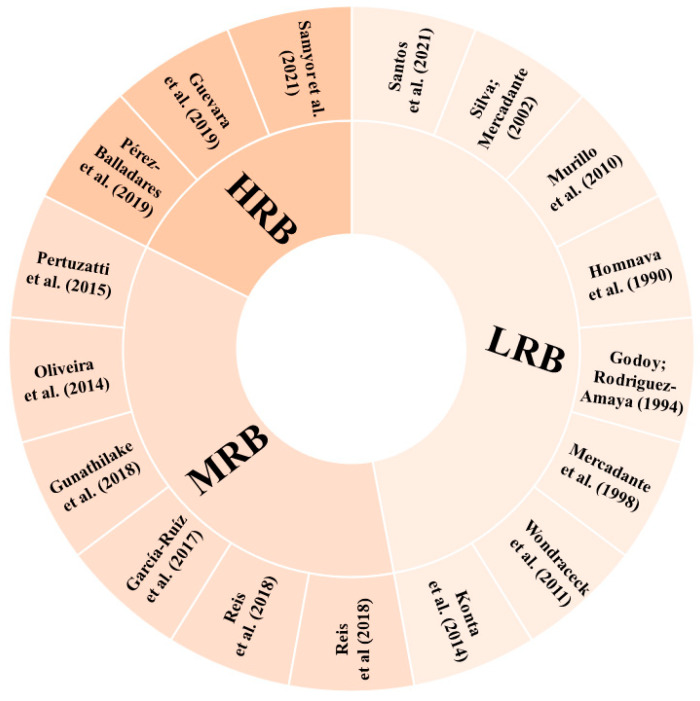
Risk of bias in each included study. LRB: low risk of bias; HRB: high risk of bias; MRB: medium risk of bias [1,2,48,49,50,51,52,54,55,56,57,58,59,60,61,62,63].

According to the parameters determined for the risk of bias in the identification of carotenoids, eight studies (47.06%) showed low risk of bias (LRQ), six studies (35.29%) showed moderate risk of bias (MRB), and three studies (17.65%) showed high risk of bias (HRB) (Figure 5). Some studies did not present the methods applied for the carotenoid analysis in a way that they could be reproduced. In cases where it was not possible to clearly identify whether or how some step was performed, the parameter was considered as “unclear”. In addition, for articles that referenced to another study without a brief or explicit description, it was considered that the authors performed the experiment under the same conditions as described in the cited reference.

The studies classified as HRB did not present, including either methodology or results, any of the parameters considered for carotenoid identification in their samples. One of these studies [63] mentioned the monitoring of the wavelengths 292 and 325 nm for β-carotene detection, not typically employed for this purpose. This lack of essential details makes it challenging for readers to replicate the study with confidence and fully understand the research.

Four studies classified as MRB [48,49,54,62] compared the compounds’ retention times with those of the respective standards available to identify the molecules. Three [48,49,62] of these studies reported the use of an UV–Vis detector, which in fact limits the number of parameters that can be applied, and one [54] had an HPLC-DAD detector, which also allows the analysis of the UV–Vis spectra characteristics. The last two studies classified as MRB [50,61] applied HPLC-DAD for carotenoid separation and identification and used the comparison with the retention time and UV–Vis spectra characteristics of the respective standards to identify the carotenoid molecules. Phytoene and phytofluene were tentatively identified by Oliveira et al. [61] according to their UV–Vis spectra characteristics. The identification of carotenoids using only the retention time or the UV–Vis spectrum and the elution order on the HPLC column can lead to misidentification, since these parameters can vary, and more than one carotenoid can present the same values, leading to confusion in the peak identification. Thus, when other parameters, such as mass spectra, are not available, it is important to carry out co-chromatography with standards [105].

A relevant and common point for all the studies classified as MRB and HRB, influencing their final score in relation to the risk of bias, was the absence of geometric isomer differentiation in the molecules tentatively identified. Moreover, none of these studies presented figures of chromatograms or spectra, and whereas there is no intention to discuss herein the total researchers’ autonomy to choose the better way to present their data and to question the peer review process, this type of figure does provide readers with valuable insights on the compound characterisation in papers intended to do so. Thus, it was considered a quality parameter in this review, even though the authors agree that it may be somehow subjective.

In the studies that demonstrated a low risk of bias, it is noteworthy that they provided details about the parameters employed for compound identification, offering a more extensive range of parameter combinations (retention time and UV–Vis and MS spectra characteristics compared with standards and available literature), besides making the information of chromatographic and spectral characteristics accessible to the readers. Besides identification tables and the description in the text of the characteristics used for carotenoid assignment, among the eight studies classified as LRB, only two [58,60] did not present pictures of their chromatograms or spectra. Three studies classified as LRB [55,56,60] did not differentiate between *E*/*Z* molecules.

The poor description of the methodology mentioned above increased the associated risk in the carotenoid assignment as assessed in this review and shown in Figure 5. Besides the description of sample preparation, extraction, identification, and quantification parameters, another important aspect of data reliability and quality that was overall overlooked in the included papers involves method validation. The validation of analytical methods refers to the application of a set of tests that analyses and documents the performance attributes of a method, demonstrating whether the method in question is suitable for obtaining the desired analytical response. It is therefore recommended that studies prioritise the use of validated methods [112].

As previously described, most of the studies included in this review used classical methods, previously validated and consolidated for carotenoid analysis. Although the long practice of successful use of a given analytical method on a variety of analytes and matrices allows for certain reliability, it is reasonable to assume the necessity of verifying the validated method when implementing it in a specific laboratory [112]. It is important to note that the specific requirements for partial validation may vary depending on local regulatory guidelines or other specific needs [113]. For instance, the Brazilian regulatory guidelines [114] advocate partial validation including at least the parameters of precision, accuracy, and selectivity. Thompson et al. (2002), in the harmonised guidelines from IUPAC [112], considered that the extent to which a laboratory needs to perform validation of a method depends on the method’s current status and the laboratory’s competence. This document recommends the reporting of precision, matrix variation, ruggedness, and linearity in the case of when the “method has been published in the scientific literature together with some analytical characteristics” [112]. The Harmonised Tripartite Guideline [115] recommends the analysis of selectivity, range and linearity, accuracy, precision, LOD, and LOQ as minimum parameters for method validation. According to Petry and Mercadante (2019), for carotenoid analysis by HPLC-DAD-MS, the following validation parameters are the most commonly applied: accuracy, precision, limit of detection, and limit of quantification [96].

The papers included presented only specific figures of validation, many of them not associated with the minimum requirements of the test according to validation guides or references. Since losses can occur during all the steps of carotenoid analysis, from extraction onwards, it is important to present the precision parameter to demonstrate the congruence of the values found [116]. In the included studies, precision was mostly verified as repeatability, i.e., by intra-assay precision with standard deviations (in general *n* = 3, Table 2), with only two studies presenting the coefficient of variation (CV) of their assays [2,62]. The CV values were below 3.7% and 8% for extractions of Silva and Mercadante (2002) [2] and Pertuzatti et al. (2015) [62], respectively. For accuracy assessment, recovery rates that are also applied for accuracy purposes [116] were reported in a single study by Homnava et al. (1990) [56]. These authors found recovery rates for β-carotene and α-carotene of 87 ± 21.4% and 82 ± 22.0%, respectively, after adding these standards to the sample matrix before extraction and saponification.

Considering the analysis of carotenoids in complex matrices like foods, matrix effect analysis can be potentially significative, but none of the studies included in this review reported this data. The matrix effect occurs due to the presence of components in the food matrix that can hamper the analysis of the desired analyte [113], which can have qualitative or quantitative consequences [117]. In analysis of non-saponified carotenoid extracts by LC-MS, triacylglycerols of the matrix can co-elute with carotenoid molecules and suppress their ionisation in MS, hampering their correct identification [96] and potentially the quantification. This is one of the reasons why LC-DAD has been more applied for carotenoid ester quantification than LC-MS, as exemplified by the single article included in this review analysing non-saponified extracts [1]. Nonetheless, when the matrix effect is not known, the method of standard addition to a blank matrix can be more satisfactory for quantification [117,118]. A close concept to the matrix effect is selectivity, which can be assessed through the interplay between the analyte and the matrix, along with an analytical curve generated without the matrix [96]. If these curves run in parallel, indicating no deviation, the determination of the substance of interest is unaffected by matrix effects, thus rendering the method quantitatively selective [96]. None of the studies included presented such data. In terms of qualitative analysis, the reliability of peak assignment and its purity, the identity verification can be further analysed by other means, including confirmatory techniques, such as derivatization reactions; also, the peak purity can be determined by tandem MS analysis, which enables the identification of impurities within the peak [106]. In the studies included in this review, derivatization reactions were applied in three studies [57,58,59], and three studies applied MS analysis [1,58,60], as can be seen in Table 1 and Section 2.2.2.

The Limit of Detection (LOD) was reported by four studies [48,49,55,62], and Limit of Quantification (LOQ) measurements were reported in three studies [48,49,62]. Finally, linearity was assessed in the studies that quantified their carotenoids using external calibration curves, none of them presenting any parameter of linearity testing besides the determination coefficient.

Although impacting the overall quality, the absence of information on the validation of the methods applied in the included papers does not mean that it was not carried out. It is possible that this detailed information was not provided for reasons of text length, prioritising the information to be presented in the paper. Nonetheless, the description of at least a few parameters of method validation or verification is warranted and another gap identified by the present research in carotenoid analysis in *Passiflora*.

Many studies included in this review reported only a few carotenoid molecules rather than a complete profile. Some characteristics of these studies may have contributed to not reporting the complete carotenoid profile of their *Passiflora* samples: 1. The identification of carotenoids was only one of the aims of the paper, as in the study by Konta et al. (2014) [60], which aimed to evaluate the antihypertensive effect of *P. edulis* pulp in rats; 2. *Passiflora* was only one of their samples, as with Guevara et al. (2019) [52] and Pérez-Balladares et al. (2019) [51], who provided analysis of 19 fruits and 13 different foods, respectively. Major carotenoids were likely highlighted, but it is worth noting that, although the activities are generally attributed to compounds more concentrated in plant samples, minor compounds could also have significant potential and potentially high efficiencies, either individually or through their synergistic interactions with other molecules. Overall, the average number of carotenoids identified per study included in this review was low, pointing to a need for a more detailed exploration of carotenoids in future research. It is known that the identification of carotenoids is a complex task that requires time and the combined information provided by specific tools and that some studies had equipment limitations, which limits the amount of information available for peak identification and increases the dependence on standards for comparison. Nonetheless, more attention should be directed to the comprehensive identification of carotenoids of various parts of *Passiflora* plants from different species, with the observation of the analytical steps and the use of more sensitive and accurate techniques.

## 3. Methodology

### 3.1. Search Strategy and Eligibility Criteria

The review was based on Preferred Reporting Items for Systematic Reviews and Meta-Analyses (PRISMA) guidelines [119]. An automated literature search, no time restriction to May 2022, on seven scientific databases, namely MEDLINE (PubMed), Web of Science, Scielo, Science Direct, SCOPUS, SpringerLink, and Wiley Online Library was performed. The search terms applied in the research with the Boolean operator “or” were i. “carotenoids” or “carotenes” or “xanthophylls”, ii. “composition” or “identification” or “characterization”, and iii. “*Passiflora*” or “passion fruit”. Three reviewers conducted searches on databases using different combinations of the chosen keywords and, after removing the duplicates, independently analysed the title and abstracts to exclude the studies that did not meet the inclusion criteria. The coordinator resolved the disagreements. All procedures were conducted blindly in the software Rayyan^®^ [32] used for systematic reviews.

Complete and original articles that carried out the separation and identification of one or more carotenoids in different parts of plants of the genus *Passiflora*, written in English, Portuguese, or Spanish were included in this review. The quantification of carotenoids could or could not have been performed. Books, book chapters, conference abstracts, review articles, and dissertations and theses were excluded, as well as original articles that did not separate or isolate carotenoids.

### 3.2. Data Synthesis

The following information was collected from the studies included for analysis: *Passiflora* species, plant part analysed, methods used for carotenoid extraction, separation, identification and quantification, carotenoid profile, and individual content, when available. Two reviewers summarised the collected information in a standardised table, in which the content was checked by experts.

### 3.3. Risk of Bias

For the analysis of the risk of bias in carotenoid identification in the studies included in this review, we used as a model the criteria applied by Gadioli et al. (2018) [4], which was adapted from the Metanalysis of Statistics Assessment and Review Instrument (MASTARI) protocol [120]. In this sense, nine criteria were established, comprising (1) Does the study describe the sampling procedure? (2) Is the extraction method described so that it can be reproduced? (3) Does the study describe the methods applied for carotenoid separation, identification, and/or quantification? (4) Were the chromatographic characteristics used as a parameter for carotenoid identification? (5) Were the UV–Vis spectra characteristics measured and used as a parameter for carotenoid identification? (6) Were the MS characteristics determined and used as a parameter for carotenoid identification? (7) Were co-chromatography or comparison with available standards applied in the carotenoid identification process? (8) Does the study present chromatograms and/or mass spectra figures? (9) Does the peak assignment contemplate the geometric configuration of cdb (*E*/*Z* isomerism)? Each criterion was evaluated as “yes”, “no”, or “unclear”, applied when the parameter was not clearly stated in the text. The frequency of “yes” was applied as a criterion to analyse the risk of bias, classifying the studies as low risk of bias (LRB) (≥70% yes), moderate risk of bias (MRB) (50–69% yes), and high risk of bias (HRB) (<50% yes) [4].

## 4. Conclusions

In conclusion, this review showed that research on carotenoids in the *Passiflora* genus remains limited, highlighting a knowledge gap. The predominant focus on *P. edulis*, despite the vast diversity within the genus, underlines a need to broaden research to include wild, and underutilised species. Such expansion is vital not only for understanding the potential health benefits of these fruits and their sustainable use but also for exploring their role in climate resilience and food security.

The analytical approach in existing studies has often lacked specificity and accuracy, with advanced techniques like MS/MS and NMR employed in only a minority of cases. This calls for a shift towards more advanced methods to accurately identify the wide array of carotenoids in *Passiflora*. The pulp has been the most examined part, with β-carotene, lutein, β-cryptoxanthin, and zeaxanthin being the most frequently identified carotenoids. Moreover, the high frequency of ζ-carotene identification suggests that it is a significant component of *Passiflora* chemical profile. However, a more comprehensive profiling, encompassing the native carotenoid profile and the analysis of other botanical parts and diverse species, is essential.

Future research should not only aim to delineate the carotenoid profiles more thoroughly but also consider the broader implications of these findings. Expanding our understanding of *Passiflora* carotenoids can lead to optimised cultivation and utilization of these species, offering economic benefits to producers and productive regions; contributing to public health; and enhancing industry applications, such as their use as food additives, in the design of functional foods, and incorporation in nutraceuticals and cosmetics.

## Figures and Tables

**Table 1:** Methods applied for extraction, separation, identification, and quantification of carotenoids from different botanical parts of *Passiflora* plants as described in the selected papers.

Sample		Extraction		Pre-Chromatographic or Pre-HPLC Steps		HPLC Separation		Identification	Quantification	Ref.
*Passiflora* Species	Plant Part	Solvent	Method	Saponification	Other Methods	Column	Mobile Phase			
*P. edulis* (yellow and purple)	Pulp	Hexane containing 0.01% BHT	Homogenization (blender)	Saturated KOH, overnight, at room temperature	Not performed	C_18_ (5 µm, 250 × 4.6 mm, Zorbax)	ACN:CH_2_Cl_2_: 0.001%TEA in MeOH (350:150:1) Isocratic Flow rate: 1.0 mL/min	HPLC-UV–Vis and HPLC-DAD	DAD, using external calibration curves	[56]
*P. edulis*	Pulp	Acetone	Homogenization (blender)	Not performed	OCC with MgO:HyfloSupercel^®^ (1:2, *w*/*w*) and elution with petroleum ether and acetone. Recromatographed on Ca(OH)_2_ column to separate *E*/*Z* isomers	C_18_ (5 µm, 250 × 4.6 mm, Vydac)	MeOH:H_2_O (98:2) Isocratic Flow rate: unspecified	HPLC-DAD	UV–VIS spectrophotometer, using the absorption coefficients of carotenoids	[57]
*P. edulis* flavicarpa	Pulp	Acetone	Maceration (mortar and pestle)	Methanolic KOH (10%), overnight	OCC with alumina and elution with either petroleum ether, or diethyl ether in petroleum ether, or ethanol in ether	Carotenes: C_18_ (5 µm; 250 × 4.6 mm, Vydac) Xanthophylls: Nitrile (5 µm; 250 × 4.6 mm, Nucleosyl)	Carotenes: MeOH Xanthophylls: EtOAc:hexane (20:80) Isocratic Flow rate: 1.0 mL/min	HPLC-DAD-MS/MS (quadrupole analyser) NMR for prolycopene	Not performed	[58]
*P. edulis* flavicarpa	Pulp	Acetone	Homogenization (blender)	Methanolic KOH (10%), overnight, at room temperature	Not performed	C_18_ (4 µm; 300 × 3.9 mm, Nova-Pak) at 29 °C	ACN:MeOH:EtOAc (75:15:10) Isocratic Flow rate: 1.0 mL/min	HPLC-DAD	DAD, using external calibration curves	[2]
*P. edulis*	Edible parts	Acetone containing NaHCO_3_	Maceration (mortar and pestle)	Methanolic KOH (5%), under N_2_ atmosphere, 2 h	Not performed	C_18_ (5 µm, 250 × 4.6 mm)	ACN:dichloromethane:MeOH (82:13:5) Isocratic Flow rate: 1.5 mL/min	HPLC-DAD	DAD, using external calibration curves	[55]
*P. cincinnata* *P. nitida* *P. setacea* *P. edulis* (yellow and purple)	Pulp	Acetone containing BHT	Maceration (mortar and pestle)	Methanolic KOH (10%), overnight, at room temperature	Not performed	C_18_ (3 µm, 150 × 4.6 mm, Waters)	0.05%TEA in ACN:MeOH:EtOAc Gradient Flow rate: 0.5 mL/min	HPLC-DAD + TLC	DAD, using external calibration curves	[59]
*P. edulis*	Pulp	Acetone	Maceration (mortar and pestle)	Methanolic KOH (10%), overnight, at room temperature	Not performed	C_30_ ^#^ (3 µm, 250 × 4.6 mm) at 22 °C	0.1% TEA in MeOH:MTBE Gradient Flow rate: 0.9 mL/min	HPLC-DAD-MS/MS (ion-trap analyser)	DAD, using external calibration curves	[60]
*P. edulis* f. flavicarpa	Pulp	Saturated NaCl:hexane (2:1); dichloromethane; EtOAc	Mixing and centrifugation	Not performed	Not performed	C_30_ (3 µm, 250 × 4.6 mm, YMC) at 30 °C	MeOH and MTBE Gradient Flow rate: 1.4 mL/min	HPLC-DAD MIR NIR	DAD, internal standardization	[61]
*P. edulis*	Pulp	Acetone + petroleum ether	Mixing and centrifugation	Ethanolic KOH (1.5 N), overnight	Not performed	C_18_ (4 µm, 150 × 4.6 mm, Shim-pak)	MeOH:ACN (30:70), MeOH:ACN:EtOAc (10:80:10), and MeOH:ACN:EtOAc (5:80:15) Gradient Flow rate: 1.0 mL/min	HPLC-UV–Vis	UV–Vis, using external calibration curves	[62]
*P. mollissima*	Pulp	MeOH:H_2_O (60:40, *v*/*v*), followed by dichloromethane	Vortex and ultrasonic bath	Not performed	Not performed	C_18_ (2.7 µm, 50 × 4.6 mm, Agilent) at 28 °C	ACN:MeOH:EtOAc Gradient Flow rate: 1.0 mL/min	RRLC-DAD	DAD, using external calibration curves	[50]
*P. edulis*	Leaves	Acetone	Maceration (mortar and pestle)	Not performed	Not performed	C_8_ (5 µm, 250 × 4 mm, Lichrospher	ACN:MeOH:CH_2_Cl_2_ (60:20:20) containing 0.1% ammonium acetate Isocratic Flow rate: 1.0 mL/min	HPLC-DAD	DAD, using external calibration curves	[54]
*P. edulis* s. flavicarpa *P. edulis* s. *edulis* *P. caerulea*	Pulp and peel	Acetone	Maceration (mortar and pestle)	Methanolic KOH (10%), overnight	Not performed	C_30_ (3 µm, 250 × 4.6 mm, YMC) at 33 °C	H_2_O:MeOH:MTBE Gradient Flow rate: 1.0 mL/min	HPLC-UV–Vis	UV–Vis, using external calibration curves	[49]
*P. caerulea*	Pulp	Acetone	Maceration (mortar and pestle)	Methanolic KOH (10%), overnight	Not performed	C_30_ (3 µm, 250 × 4.6 mm, YMC) at 33 °C	H_2_O:MeOH:MTBE Gradient Flow rate: 1.0 mL/min	HPLC-UV–Vis	UV–Vis, using external calibration curves	[48]
*P. quadrangularis*	Edible parts	Acetone	Maceration (mortar and pestle)	Methanolic KOH (10%) overnight	Not performed	C_18_ (5 µm, 250 × 4.6 mm, Eclipse Plus)	MeOH:isopropanol (35:65) Isocratic Flow rate: 1.0 mL/min	HPLC-DAD	DAD, using external calibration curves	[52]
*P. ligularis* Juss. *P. mollissima*	Edible parts	Chloroform	Agitation	Methanolic KOH (5%), for 4 h at 50 °C	Not performed	C_18_ (5 µm, 250 × 4.6 mm, Eclipse Plus)	MeOH:isopropanol (35:65) Isocratic Flow rate: 1.0 mL/min	HPLC-DAD	DAD, using external calibration curves	[51]
*P. edulis* Sims	Pulp	MeOH	Homogenization and centrifugation	Not performed	Not performed	C_18_ (5 µm, 250 × 4.6 mm, unspecified brand)	MeOH:ACN (20:80) Isocratic Flow rate: 0.8 mL/min	HPLC-UV–Vis	Unclear	[63]
*P. tenuifila*	Whole fruit	Acetone	Maceration (mortar and pestle)	Not performed	Not performed	C_30_ (5 µm, 250 × 4.6 mm, YMC)	MeOH:MTBE:H_2_O Gradient Flow rate: 1.0 mL/min	HPLC-DAD-MS/MS (ion-trap analyser)	DAD, using external calibration curves	[1]

HPLC: high-performance liquid chromatography; UV–Vis: UV–visible detector; DAD: diode array detector; OCC: open column chromatography; TLC: thin-layer chromatography; MS/MS: tandem mass spectrometry; RRLC: rapid resolution liquid chromatography; NaCl: sodium chloride; BHT: butylated hydroxytoluene; KOH: potassium hydroxide; ACN: acetonitrile; CH_2_Cl_2_: methylene chloride; TEA: triethylamine; MeOH: methanol; H_2_O: water; EtOAc: ethyl acetate; MIR: mid-infrared spectroscopy and NIR: near-infrared spectroscopy; ^#^ The information about the parameters used in the HPLC-DAD-MS/MS analyses was not expressly written in the article and was then collected in the article cited as reference [64].

**Table 2:** Carotenoids identified and quantified in different parts and species of *Passiflora* plants as reported by the papers selected in this review.

Sample		Carotenoid Profile ^1^	Carotenoid Content														Ref.
*Passiflora* Species	Plant Part																
*P. edulis* (yellow and purple)	Pulp		µg/g sample fw														[56]
			Yellow 1				Yellow 2				Purple 1				Purple 2		
		β-carotene	7.50 ± 0.36				3.00 ± 1.40				3.36 ± 0.02				11.50 ± 0.9		
		α-carotene	0.70 ± 0.08				nd				nd				nd		
		β-cryptoxanthin	0.53 ± 0.03				0.40 ± 0.11				0.42 ± 0.006				0.40 ± 0.13		
*P. edulis*	Pulp		µg/g sample fw														[57]
		(all-*E*)-β-carotene	4.70 ± 1.00														
*P. edulis* flavicarpa	Pulp		µg/g sample fw														[58]
		Phytoene	Not quantified														
		Phytofluene															
		β-carotene															
		ζ-carotene															
		Prolycopene															
		Neurosporene															
		Lycopene															
		Monoepoxi-β-carotene															
		β-cryptoxanthin															
		β-citraurin															
		Antheraxanthin															
		Violaxanthin															
		Neoxanthin															
*P. edulis* flavicarpa ^2^	Pulp									µg/g sample fw							[2]
			Batch 1					Batch 2		Batch 3			Batch 4			Batch 5	
		β-cryptoxanthin	0.69 ± 0.02					0.45 ± 0.01		1.27 ± 0.04			1.75 ± 0.03			2.65 ± 0.09	
		Prolycopene	1.71 ± 0.03					2.25 ± 0.06		0.30 ± 0.00			3.02 ± 0.07			2.59 ± 0.04	
		Neurosporene	tr					tr		nd			nd			nd	
		Mixture ^3^	3.69 ± 0.03					4.59 ± 0.02		0.63 ± 0.02			3.34 ± 0.04			2.91 ± 0.08	
		γ-carotene	nd					nd		Tr			nd			nd	
		(*Z*)-ζ-carotene	4.45 ± 0.07					2.82 ± 0.06		0.74 ± 0.02			7.38 ± 0.07			2.59 ± 0.04	
		ζ-carotene	7.78 ± 0.21					12.86 ± 0.46		1.26 ± 0.04			3.93 ± 0.08			3.05 ± 0.08	
		β-carotene	4.48 ± 0.07					2.39 ± 0.03		10.79 ± 0.40			6.77 ± 0.12			13.35 ± 0.31	
		(13*Z*)-β-carotene	tr					tr		0.36 ± 0.01			tr			tr	
		Phytoene	nq					nq		nq			nq			nq	
		Phytofluene	nq					nq		nq			nq			nq	
*P. edulis*	Edible parts		µg/g sample fw														[54]
		Lutein	0.1 ± 0.1														
		Zeaxanthin	0.2 ± 0.1														
*P. cincinnata* *P. nitida* *P. setacea* *P. edulis* (native yellow and purple and commercial a and b)	Pulp		µg/g sample fw														[59]
			*P. cincinnata*	*P. nitida*		*P. setacea*			*P. edulis* n.y			*P. edulis* n.p		*P. edulis*c.a		*P. edulis*c.b	
		Neoxanthin	nq	nd		nd			nd			nd		nq		nq	
		Antheraxanthin	nq	nq		nq			nq			nq		nq		nq	
		Lutein	nq	nd		nq			nd			nd		nd		nd	
		Zeaxanthin	nd	nd		nq			nq			nd		nq		nq	
		Phytofluene	nd	nd		nd			nq			nq		nq		nq	
		(all-*E*)-violaxanthin	tr-0.02 ± 0.00	nd		tr			0.50 ± 0.05			nd		0.60 ± 0.08		0.50 ± 0.10	
		(*Z*)-violaxanthin	nd	nd		0.18 ± 0.06			nd			nd		1.20 ± 0.16		1.21 ± 0.20	
		β-cryptoxanthin	nd	nd		nd			0.24 ± 0.02			0.20 ± 0.03		1.75 ± 0.08		1.80 ± 0.20	
		Prolycopene	nd	nd		nd			3.03 ± 0.08			5.90 ± 0.50		5.43 ± 0.18		0.87 ± 0.09	
		Poly-(*Z*)-carotene	nd	nd		nd			1.30 ± 0.10			3.40 ± 0.20		4.93 ± 0.18		1.27 ± 0.06	
		(*Z*)-ζ-carotene	nd	nd		tr			6.28 ± 0.15			12.10 ± 0.70		6.83 ± 0.25		2.00 ± 0.10	
		(all-*E*)-ζ-carotene	nd	nd		nd			5.40 ± 0.28			10.95 ± 0.30		11.40 ± 0.40		2.30 ± 0.10	
		(all-*E*)-β-carotene	0.03–0.06 ± 0.00–0.01	0.005 ± 0.00		0.66 ± 0.09			2.84 ± 0.06			2.60 ± 0.10		3.60 ± 0.10		7.80 ± 0.80	
		(13*Z*)-β-carotene	nd	nd		0.08 ± 0.00			0.38 ± 0.08			tr		0.40 ± 0.00		0.37 ± 0.02	
*P. edulis* f. flavicarpa	Pulp		µg/g sample fw														[60]
		ζ-carotene	16.7 ± 0.3														
		β-carotene	13.8 ± 0.2														
*P. edulis* f. flavicarpa	Pulp		µg/g sample dw														[61]
		β-carotene	60.00 ± 60.00														
		Phytoene	50.00 ± 30.00														
		Phytofluene	20.00 ± 10.00														
*P. edulis* f. flavicarpa	Pulp		Organic *								Conventional *						[62]
		Lutein + Zeaxanthin	0.01								0.01						
		β-cryptoxanthin	139.40								249.90						
		Lycopene	0.02								0.28						
		β-carotene	0.56								0.77						
*P. mollissima*	Pulp		µg/g sample dw														[50]
		α-carotene	1.64 ± 0.50														
		β-carotene	79.74 ± 30.38														
		Zeaxanthin	1.86 ± 0.49														
*P. edulis*	Leaves		µg/g sample dw														[54]
		β-carotene	240.00 ± 5.00														
		Lutein	240.00 ± 1.00														
*P. edulis* S. flavicarpa, *P. edulis* S. edulis, *P. caerulea*	Pulp and peel		µg/g sample dw														[49]
			Pulp							Peel							
			*P. edulis* Sims flavicarpa		*P*. *edulis* Sims edulis			*P*. *caerulea*		*P*. *edulis* Sims flavicarpa			*P. edulis* Sims *edulis*			*P.* *caerulea*	
		Lutein	0.44 ± 0.02		0.11 ± 0.001			1.05 ± 0.03		5.05 ± 0.25			3.67 ± 0.18			28.81 ± 1.49	
		Zeaxanthin	0.66 ± 0.01		0.75 ± 0.001			0.91 ± 0.02		0.66 ± 0.002			0.49 ± 0.03			3.24 ± 0.11	
		Cryptoxanthin	2.54 ± 0.03		0.31 ± 0.001			nd		0.75 ± 0.001			0.75 ± 0.001			6.17 ± 0.38	
		α-carotene	0.86 ± 0.05		0.68 ± 0.02			nd		nd			0.37 ± 0.01			4.20 ± 0.15	
		β-carotene	13.34 ± 0.79		1.72 ± 0.02			7.44 ± 0.16		2.73 ± 0.12			7.16 ± 0.31			212.74 ± 6.76	
		Lycopene	nd		nd			44.05 ± 1.35		nd			nd			nd	
*P. caerulea*	Pulp (juice)		sample fw ^#^														[48]
		Lutein	8.59 ± 0.41														
		Zeaxanthin	10.20 ± 0.25														
		β-cryptoxanthin	35.33 ± 1.60														
		α-carotene	7.02 ± 0.26														
		β-carotene	37.98 ± 1.78														
		Lycopene	108.39 ± 3.29														
*P. quadrangularis*	Edible parts		µg/100 g sample fw *														[52]
		β-carotene	<5.0														
		Lycopene	nd														
		Lutein	nd														
*P. ligularis* Juss. *P. mollissima*	Edible parts		µg/g sample fw														[51]
			*P. ligularis* Juss							*P. mollissima* (Kunth) L.H. Bailey							
		β-carotene	1.68 ± 0.26							16.25 ± 2.17							
		Lutein	2.56 ± 0.48							45.37 ± 0.47							
*P. edulis* Sims	Pulp		µg/g sample fw *														[63]
			Fresh pulp							Pulp powder							
		β-carotene	117.90							132.60							
*P. tenuifila*	Whole fruit							µg/g sample fw									[1]
			Batch 1					Batch 2					Batch 3				
			Ripe					Mature-green			Ripe		Mature-green			Ripe	
		(all-*E*)-violaxanthin	1.54 ± 0.02					3.20 ± 0.07			1.43 ± 0.04		3.05 ± 0.18			1.86 ± 0.09	
		Not identified mixture	1.50 ± 0.05					1.75 ± 0.08			1.47 ± 0.06		1.63 ± 0.06			1.66 ± 0.06	
		(all-*E*)-antheraxanthin	1.69 ± 0.03					2.21 ± 0.08			1.63 ± 0.06		2.10 ± 0.09			2.01 ± 0.11	
		(13*Z*)-lutein and/or (13′*Z*)-lutein	1.45 ± 0.01					1.61 ± 0.02			1.47 ± 0.04		1.57 ± 0.04			1.60 ± 0.06	
		(all-*E*)-lutein	6.93 ± 0.65					13.24 ± 0.30			5.52 ± 0.12		11.86 ± 0.90			10.09 ± 0.52	
		(all-*E*)-zeaxanthin	2.97 ± 0.04					2.44 ± 0.05			2.46 ± 0.15		2.10 ± 0.08			2.38 ± 0.06	
		(9*Z*)-lutein	1.41 ± 0.01					1.50 ± 0.01			1.42 ± 0.01		1.50 ± 0.02			1.48 ± 0.02	
		(all-*E*)-violaxanthin-myristate	1.39 ± 0.03					1.42 ± 0.02			1.43 ± 0.02		1.40 ± 0.03			1.47 ± 0.01	
		Not identified carotenoid-myristate	1.46 ± 0.01					1.52 ± 0.02			1.44 ± 0.03		1.47 ± 0.02			1.48 ± 0.01	
		(all-*E*)-violaxanthin-palmitate	1.36 ± 0.03					1.49 ± 0.01			1.44 ± 0.02		1.41 ± 0.01			1.46 ± 0.01	
		(13*Z*)- or (15*Z*)-β-carotene	nd					nd			1.84 ± 0.04		nd			1.73 ± 0.09	
		(all-*E*)-luteina-3′-*O*-myristate	1.69 ± 0.07					1.54 ± 0.02			1.75 ± 0.06		1.53 ± 0.02			1.78 ± 0.02	
		(all-*E*)-β-carotene	8.38 ± 0.11					10.95 ± 0.51			8.38 ± 0.48		9.78 ± 0.84			11.10 ± 0.17	
		(9*Z*)-β-carotene	1.80 ± 0.05					1.92 ± 0.02			2.10 ± 0.12		1.82 ± 0.06			2.07 ± 0.05	
		Not identified	1.70 ± 0.02					1.46 ± 0.01			1.49 ± 0.01		1.41 ± 0.02			1.64 ± 0.01	
		(all-*E*)-lutein-dimyristate	1.44 ± 0.02					1.38 ± 0.01			1.43 ± 0.01		1.32 ± 0.01			1.42 ± 0.01	
		Not identified	1.44 ± 0.01					nd			1.36 ± 0.03		nd			1.32 ± 0.08	

^1^ The nomenclature of carotenoids presented in this table has been kept identical to that reported in the respective articles. ^2^ Batch separated by time of fruit acquisition (April to July 1999). ^3^ Mixture of carotenoids that co-eluted during the analyses; fw: fresh weight; dw: dry weight; nq: detected but not quantified, i.e., below the quantification limit; nd: not detected; tr: traces; n.y: native yellow; n.p: native purple; c.a: commercial a; c.b: commercial b. * Studies did not report the standard deviation (SD) of their values. ^#^ The study did not specify the measurement unit.

## Data Availability

No new data were created or analysed in this study. Data sharing is not applicable to this article.
